# Estimating Age Ratios and Size of Pacific Walrus Herds on Coastal Haulouts using Video Imaging

**DOI:** 10.1371/journal.pone.0069806

**Published:** 2013-07-31

**Authors:** Daniel H. Monson, Mark S. Udevitz, Chadwick V. Jay

**Affiliations:** United States Geological Survey, Alaska Science Center, Anchorage, Alaska, United States of America; Université de Sherbrooke, Canada

## Abstract

During Arctic summers, sea ice provides resting habitat for Pacific walruses as it drifts over foraging areas in the eastern Chukchi Sea. Climate-driven reductions in sea ice have recently created ice-free conditions in the Chukchi Sea by late summer causing walruses to rest at coastal haulouts along the Chukotka and Alaska coasts, which provides an opportunity to study walruses at relatively accessible locations. Walrus age can be determined from the ratio of tusk length to snout dimensions. We evaluated use of images obtained from a gyro-stabilized video system mounted on a helicopter flying at high altitudes (to avoid disturbance) to classify the sex and age of walruses hauled out on Alaska beaches in 2010–2011. We were able to classify 95% of randomly selected individuals to either an 8- or 3-category age class, and we found measurement-based age classifications were more repeatable than visual classifications when using images presenting the correct head profile. Herd density at coastal haulouts averaged 0.88 walruses/m^2^ (std. err. = 0.02), herd size ranged from 8,300 to 19,400 (CV 0.03–0.06) and we documented ∼30,000 animals along ∼1 km of beach in 2011. Within the herds, dependent walruses (0–2 yr-olds) tended to be located closer to water, and this tendency became more pronounced as the herd spent more time on the beach. Therefore, unbiased estimation of herd age-ratios will require a sampling design that allows for spatial and temporal structuring. In addition, randomly sampling walruses available at the edge of the herd for other purposes (e.g., tagging, biopsying) will not sample walruses with an age structure representative of the herd. Sea ice losses are projected to continue, and population age structure data collected with aerial videography at coastal haulouts may provide demographic information vital to ongoing efforts to understand effects of climate change on this species.

## Introduction

The Pacific walrus (*Odobenus rosmarus divergens*) is a sea-ice-associated pinniped that ranges over the continental shelves of the Bering and Chukchi seas [1]. Walruses play a major ecological role in Beringia [2] and provide an important cultural and subsistence resource for coastal communities in Alaska and Chukotka [3]. Walrus life history is closely tied to sea ice, particularly for females and young because they use sea ice for most of the year. Females begin calving each spring in the Bering Sea, and then follow the retreating ice north into the Chukchi Sea where they continue to nurse young and molt. Walruses are benthic foragers, and while summering in the Chukchi Sea, females and young are found with the ice over the shallow, productive continental shelf. The ice provides a platform for resting and refuge from predators and disturbance [1]. As sea ice re-forms and advances southward in September and October, the females and young migrate back to their winter range in the Bering Sea.

The extent of summer sea ice in the Chukchi Sea has declined substantially in recent years [4], and this decline is projected to continue [5]–[8]. Recently, ice-free conditions have developed over the shelf in the Chukchi Sea in late summer/fall causing thousands of female walruses and their calves to aggregate in herds onshore in both Alaska and Chukotka [9], [10], [11]. The current and future cumulative effects of sea ice loss and associated stressors (e.g., increased vessel traffic and resource development activities) on the Pacific walrus population [12], [13], were considered by the U.S. Fish and Wildlife Service (USFWS) in their 2011 decision that listing the Pacific walrus as threatened under the Endangered Species Act was warranted but precluded due to other higher priority listing actions (FR Vol. 76, No. 28:7634-7679). The USFWS will need more information on the status of the Pacific walrus population and its response to climate change before a final listing decision in 2017.

The harsh and remote nature of the offshore arctic environment makes studying walrus populations logistically difficult, and walruses are difficult to capture and handle. Pacific walrus reproductive rates have been estimated based on examination of reproductive tracts from harvested animals [14] but survival rates have never been directly estimated for this population. Age ratios are commonly used to estimate recruitment for species with relatively stable adult populations, such as ungulates [15]–[18] and other pinnipeds [19], [20], and may be valuable for estimating walrus demographic rates. In the past, age structure information has been used in combination with other data to model Pacific walrus population dynamics [14].

Walruses are a classic K-selected species [21] whose life history includes slow growth, late maturation, low reproductive rate, extended dependency (up to 2 or more years) and high adult survival rates leading to a long life expectancy of 30 to 40 years [1]. In addition, their tusks continue to grow throughout their lives [1]. Dr. F. H. Fay exploited these characteristics to develop a method to visually classify individual walruses into eight age categories based on their head morphology and the ratio of tusk length to snout width or depth [22]–[24]. The method was previously used in several at-sea surveys in the 1980's and 1990's [23], [24].

The recent loss of summer sea ice in the Chukchi Sea, resulting in thousands of female walruses and their dependent young resting on shore, has created opportunities to apply the Fay [22], [23] aging method to large numbers of walruses at logistically accessible coastal locations. However, classifying individual walruses to sex and age using head morphology and tusk to snout ratios requires a clear frontal view or direct side view of the animal's head. Obtaining a clear view of individuals within herds at coastal haulouts is difficult because of the high density of individuals within these herds, and because the herds can occur on low relief beaches that restrict visual access at ground level. In addition, herds on shore can number in the thousands, which exposes individuals to risk of injury and death from trampling (especially calves) if the herd is disturbed [25]–[32]. Therefore, a viewing platform that provides an over-head perspective and does not pose a significant risk of disturbance is required.

We evaluated the use of Fay's [22], [23] method to estimate age ratios of walruses in large herds hauled out on the northwest coast of Alaska, using high-resolution images obtained from an image-stabilizing video system mounted on a helicopter flying obliquely to the herds at altitudes high enough to avoid disturbing the walruses. Our objectives were to assess our ability to age walruses from images taken from high altitudes, estimate age structure within and among herds, and estimate the densities and areal coverage of the herds, and thus the number of animals in the herds. We successfully aged walruses from images taken at altitudes of 915 m to 1220 m with minimal disturbance to the herd, and estimated animal density and herd coverage. Age structure and size of herds will be valuable data for use in population modeling efforts to inform management decisions. In addition, we believe the technology described here is a valuable tool and useful for making detailed observations on a variety of wildlife species inhabiting relatively open habitats where approaching closely is not possible or practical.

## Materials and Methods

### Filming

**Figure 1:**
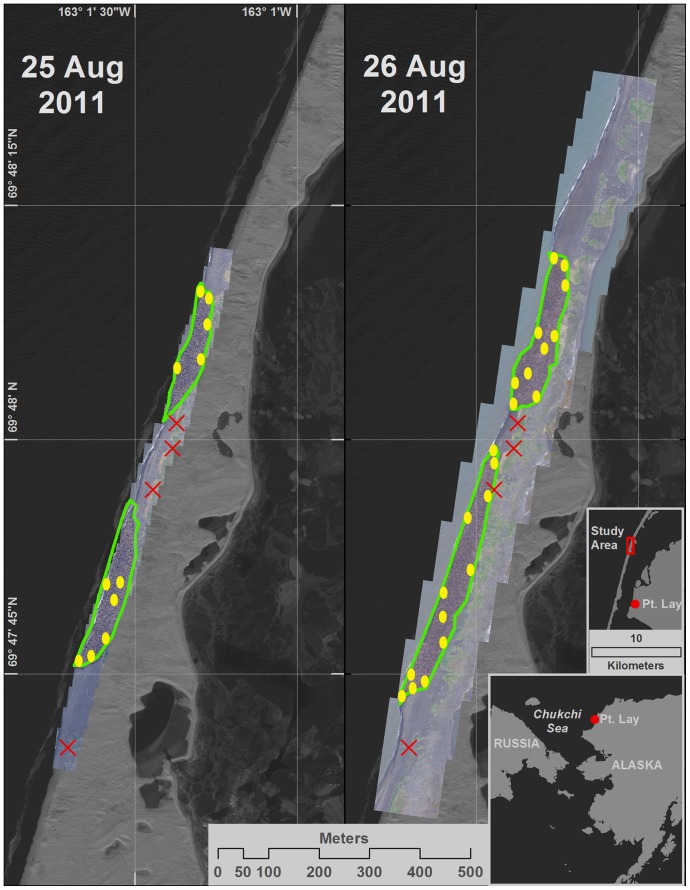
Walrus haulout location near Pt. Lay, Alaska. Equally scaled composite images of two walrus herds hauled out near Pt. Lay on the NW coast of Alaska on August 25^th^ (left photo) and August 26^th^ (right photo), 2011. Green lines delineate the outline of the herd and yellow circles identify approximate location of the focal point for hold sequences. Red X's indicate the north location of paired reference marks used to scale the images.

We recorded video footage of walrus herds hauled out near Pt. Lay, Alaska (Fig. 1) in the falls of 2010 and 2011 with a Cineflex v14 HD video system mounted on a Robinson model R44 helicopter (Zatzworks Inc. Homer, AK). The Cineflex system included a gyro-stabilized Sony HDC 1500 4∶4∶4 1080p camera equipped with a 42×9.7 telephoto lens (plus doubler), and a HDCAM SR Recorder (4∶4∶4/1080 60p). Footage was taken during filming passes flown along a north-south flight path paralleling the shoreline approximately 200 m to 400 m offshore. In 2010, filming altitude was approximately 1220 m. In 2011, we began filming at 1220 m and when conditions permitted, filmed down to approximately 915 m. While adjacent to the herd, the helicopter maintained a steady altitude and slow forward speed (∼37 to 55 km/hr), and minimized changes in speed, direction, and power. Ground-based observers noted any visible reactions to the helicopter by the walrus herd.

In 2011, we placed four pairs of reference marks on the beach near the herd to scale the images for determining herd density. Each pair of marks was located with a tape precisely 10 m apart and oriented parallel to the shoreline. We recorded the GPS location of the northern mark of each pair using the automated position averaging feature of a Garmin model map76 GPS (Garmin International, Inc., Olathe, KS). When recording a location, we allowed approximately 3–5 min until position accuracy was <3 m.

We recorded two types of video sequences: 1) “hold sequences” used for age classification, and 2) “transect sequences” used to estimate herd density. During hold sequences, the videographer haphazardly chose a focal point within the herd on approach, and after a wide-angle establishing shot, zoomed into the chosen focal point (maximum zoom), maintained this focal point as the helicopter passed, and finished with another wide-angle view. The goal was to provide multiple perspectives from which to view the walruses during a hold sequence, increasing the likelihood that an animal exhibited a frontal or side view of its head for visually classifying its sex and age. Hold times lasted only several seconds in 2010 because we were concerned about the potential to disturb the herd as we flew by. Because the herds were not disturbed by the flights, in 2011, the hold was maintained as long as practical.

In 2011, we also recorded transect sequences to determine herd density. These sequences were necessary because the scale of the video image in hold sequences changed constantly. Transect sequences maintained the scale of the image throughout the transect by fixing the camera's zoom level and orientation on approach, and holding this camera position as the helicopter passed parallel to the herd at a constant altitude.

Video sequences were processed with the ProRes codec in Final cut-pro (Apple Inc., Cupertino, CA) or the MPEG I-Frame codec in Adobe Premiere Pro (v5.5, Adobe Systems Inc., San Jose, CA), and output as high-resolution “.mov” files. We imported the .mov files into Adobe Premiere Pro where we reviewed the video, identified beginning and end times of each sequence, and exported reference images.

### Age classification

We selected a reference frame for each hold sequence and recorded its video clock time using Adobe Premiere Pro, targeting images with the most direct over-head perspective. We created a reference image by exporting the reference frame as a “.TIFF” file, and then used the “manual tag” tool in Image-Pro Plus image analysis software (v7, Media Cybernetics, Inc., Bethesda, MD) to sequentially number (i.e., “mark”) all individuals within an area of each reference image. Images collected from lower altitudes had a relatively small field of view, and we marked all animals that were fully included in the image. Video collected from higher altitudes had larger fields of view, so we selected a strip within the image oriented parallel to the shoreline and marked all animals that were fully included in the strip.

After marking walruses in a reference image, we randomly selected a subset of 10% or 30 marked individuals (whichever was greater) for age classification. D. Monson performed all aging and classifications. If the age of a selected animal could not be determined in the reference image, we located the animal within the corresponding video reference frame, and viewed the remainder of the hold sequence until we located an image with the requisite head profile. We detected some individuals only after viewing the hold sequence because they were hidden from view by other animals in the reference image. Calves made up the vast majority of these hidden individuals. We marked these hidden individuals as we detected them, and made them available for random selection to avoid biasing calf∶cow ratios.

We always attempted to classify walruses to the 8-category aging resolution (see Figure 1 in [24]). However, for animals that did not exhibit an adequate view of the tusks and snout, this was not always possible and we classified them to a lower, 3-category age-class resolution (0–2 yrs, 3–5 yrs, and ≥6 yrs-old). In 2010, we visually classified walruses by viewing the video recordings similarly to the real-time aging methods developed by Fay [22], [23]. However, we had the advantage of being able to review the video multiple times and frame by frame. In 2011, we classified walruses only visually in the first five hold sequences. However, in the next 35 hold sequences, we expanded on the Fay [22], [23] method by defining threshold tusk∶snout ratio values that distinguished age classes (Table 1), and measured precise tusk length and snout dimensions on exported images of each randomly selected walrus using the “measure distance” tool in Image-Pro Plus (v7, Media Cybernetics, Inc., Bethesda, MD). We then classified walruses with the 8-category age classifications based on threshold values of the tusk∶snout ratios (Table 1) determined from data collected on harvested animals presented in Fay and Kelly [23]. Unless an animal was clearly a male (generally adults), we classified all individuals with the female threshold values. Calves and yearlings have no visible tusks (see Figure 1 in [24]), and we aged them on body size alone in both 2010 and 2011.

**Table 1:** Tusk∶snout ratio threshold values used for assigning age based on measurements presented in Fay and Kelly [23] and calculated as the midpoints between the average ratios for each age-class.

Sex	Age^1^ (yr)	Threshold width ratio	Threshold depth ratio
**Females**	0	---	---
	1	0.02	0.00
	2	0.10	0.09
	3	0.27	0.36
	4–5	0.45	0.66
	6–9	0.65	1.05
	10–15	0.94	1.59
	15+	1.27	2.13
**Males**	0	---	---
	1	0.02	0.00
	2	0.10	0.09
	3	0.25	0.30
	4–5	0.44	0.64
	6–9	0.69	1.15
	10–15	0.95	1.63
	15+	1.23	2.20

The width ratio is the ratio of tusk length to snout width (head-on profile) and the depth ratio is the ratio of tusk length to snout depth (side-view profile).

1 Age assigned to ratios ≥ threshold value.

We defined threshold tusk∶snout ratio values as the midpoints between the average ratios for each age-class. We assigned the older age-class to individuals with ratio values greater than or equal to a threshold value while individuals with ratio values less than the threshold were assigned to the younger age-class (Table 1). When a measureable head profile was not available, a visual classification was made based on the visual cues available. For visual classification, we also used other available cues such as the presence of a bacular ridge or skin bosses to confirm sex of males. Generally, we classified 0–2 yr-olds at least to the 3-category scale based on body size alone, regardless of the quality of the head profile view. In contrast, we categorized larger individuals as “unknown” when no views of the tusks and snout were available.

We compared estimated age structures for the portion of the herd that could be classified to the 8-category level to the portion that could only be classified to the 3-category level with a likelihood ratio chi square test [33]. We used logistic regression [34] to estimate the proportion of walruses in each 3-category age class that could be classified to the 8-category level, and used t-tests of differences between least square means to evaluate differences in these proportions among categories. For a subset of 10 sequences in 2011, we attempted both visual and measurement-based estimates of age on all individuals. We made visual estimates first and then obtained a measurement-based age determination, and we assumed the measurement-based estimate was more accurate and discarded the initial visual determination in the final analysis. We used logistic regression [34] to estimate the probability of an error as a function of the initially determined visual age class. We characterized the magnitude and direction of each error in terms of the number of age categories by which the initial visual determination was either too high or too low. We estimated the mean value of the error for each visually determined age class and used bootstrap resampling [35] to estimate the associated 95% confidence intervals. We did separate analyses for the 3-category and 8-category age determinations.

For another subset of 10 sequences in 2011, we attempted to make two measurement-based age estimates for all animals with a measurement-based age in the reference image. That is, before reviewing the hold sequence, we decided if the reference image provided a measurable head profile, and if it did we obtained a measurement-based age classification mimicking a situation where only one still image is available. We then searched the rest of the hold sequence to find a second measurable frame as good as or better than the reference frame. We considered an image better if the walrus exhibited a frontal or side view of its head more perpendicular to the camera. If the measurement-based age determination from the second image differed from the determination in the reference image, we assumed the initial determination was in error. Again, we estimated the probability of an error with logistic regression and estimated the mean error with bootstrap confidence intervals as described above, with separate analyses for 3- and 8-category age determinations.

### Spatial and temporal age distribution

For each hold sequence, we recorded the date along with a herd identifier. We also identified the focal point of the hold sequence on the scaled composite transect images (Fig. 1) and estimated its distance to the water line (in 5 meter increments). Finally, we used wide-angle views of the herd along with the scaled images to identify areas of beach occupied by walruses over the previous 24 hrs and identified each hold sequence relative to recentness of occupation (i.e., location occupied within past 24 hrs or not).

To investigate factors potentially related to the distribution of age classes among and within herds, we focused our analysis on how proportions of the dependent age classes (0–2 yr-olds) varied among hold sequences. We used a logistic mixed-effects model [34], [36] to estimate the effects of herd; date; whether or not the site of the hold sequence was occupied by walruses the previous day (recentness); position within the herd, as indexed by distance from water (distance); and the interaction between recentness and distance on whether a classified individual was in the 0–2 age-class or not. We included a random effect for hold sequence to account for correlations among walruses within the same sequence. Most hold sequences were located at one edge or completely within a herd, but some sequences spanned the entire breadth of the herd and position within the herd was not a meaningful variable for this latter set of sequences. Therefore, we categorized hold sequences based on whether they spanned the entire breadth of the herd or not and used the interaction with this classification to define the distance and distance*recentness effects in the model. This allowed us to consider only these effects for hold sequences that did not span the entire breadth of the herd. We used Type III F-tests for significance to assess fixed effects and eliminated any variables that were not significant at the α = 0.05 level to obtain final estimates of the remaining effects.

### Herd size and density

For each herd and date, we exported a set of overlapping images from transect sequences and stitched them together using the “photomerge” tool within Adobe Photoshop (Adobe Systems Inc., San Jose, CA). We georeferenced the composite image of the herd to maps of NW Alaska in ARCMap (ESRI, Redland, CA) using the GPS coordinates of the reference marks, and calculated the area of a digitized outline of the herd to estimate its areal coverage. We used the reference marks to create a scaling factor in the Image-Pro Plus image processing software, and used this scaling factor to create a 10 m×10 m grid, covering the entire image. We randomly selected 20 grid cells and counted all individuals with ≥50% of their body within each selected cell. Grid cells in the interior of the herd were always 100 m^2^ but for grid cells on the edge of the herd, we determined the area of the cell contained within the herd outline. Density within each cell was estimated as the number of walruses divided by the area of the cell within the herd outline. We used ANOVA to compare mean walrus densities among herds and days. We used ratio estimates [37:68–70] to estimate the overall density and total number of walruses in each herd on each day. We summed the ratio estimates and divided by the summed areas to obtain a stratified estimate of the combined density for all herds and days [37:118–120].

This work was conducted under the authority of the US Fish and Wildlife Service (USFWS) Marine Mammal Permit No. MA801652-6.

## Results

### Filming and marking

Walruses began hauling out on the NW coast of Alaska by early September in 2010 and by late August in 2011 (NOAA Alaska Fisheries Science Center: COMIDA Survey Project. http://www.afsc.noaa.gov/NMML/cetacean/bwasp/flights_COMIDA.php, accessed 28 June 2012). In both years, the herds formed on a barrier island near the village of Pt. Lay (Fig. 1). Filming occurred on the evening of 3 September and morning of 4 September 2010, and 24–26 August 2011. In 2010, minimizing potential disturbance was a primary concern, and we made only two passes on each flight (one going north and one returning south). In 2011, video flights lasted up to several hours and included multiple passes in both directions.

We obtained 51 hold sequences during the two field seasons (Table 2). The focal points of the hold sequences included locations along the water's edge, interior locations within the herd, and locations at various points along the landward periphery of the herd (Fig. 1). Hold times were short in 2010 (average = 3.3 sec, min = 1.5 sec, max = 7.5 sec; Table 2). In 2011 we attempted to make holds as long as practical (average = 64 sec, min = 27 sec, max = 172 sec; Table 2). In 2011, we recorded transect sequences on the 25^th^ and 26^th^ of August (Fig. 1).

**Table 2:** Number of video “hold” sequences recorded at a coastal walrus haulout site near Pt. Lay Alaska in 2010 and 2011, and number of marked and randomly selected individuals from hold sequence reference images.

Year	Herd number	Date	No. hold sequences	No. walruses marked	No. randomly selected for aging	Ave. no. marked per seq.	Ave. no. selected per seq.	Approx. filming altitude (m)	Ave. hold-time (sec.)
2010	1	9/3/2010	8	3,026	327	378	41	1,220	3.5
2010	2	9/3/2010	3	1,292	133	431	44	1,220	2.5
**2010**	**all**	---	**11**	**4,318**	**460**	**393**	**42**	---	**3.3**
2011	3	8/24/2011	3	696	99	232	33	1,070	67
2011	3	8/25/2011	7	2,163	316	309	45	915	99
2011	3	8/26/2011	11	4,134	472	376	43	1,220	55
2011	4	8/24/2011	3	527	92	176	31	1,070	58
2011	4	8/25/2011	5	1,461	200	292	40	915	80
2011	4	8/26/2011	11	4,381	501	398	46	1,220	44
**2011**	**all**	---	**40**	**13,362**	**1,680**	**334**	**42**	---	**64**
**All**	**All**	---	**51**	**17,680**	**2,140**	**347**	**42**	---	---

We “marked” 4,318 walruses in 11 reference images from 2010 and 13,362 walruses in 40 reference images from 2011 (Table 2). Overall, we marked an average of 347 walruses per image (min = 112, max = 582; Table 2). We attempted to age 460 randomly selected individuals (11% of marked) in 2010 and 1,680 randomly selected individuals (13% of marked) in 2011 (average = 42 selected per image, min = 28, max = 66; Table 2).

### Age classification

**Figure 2:**
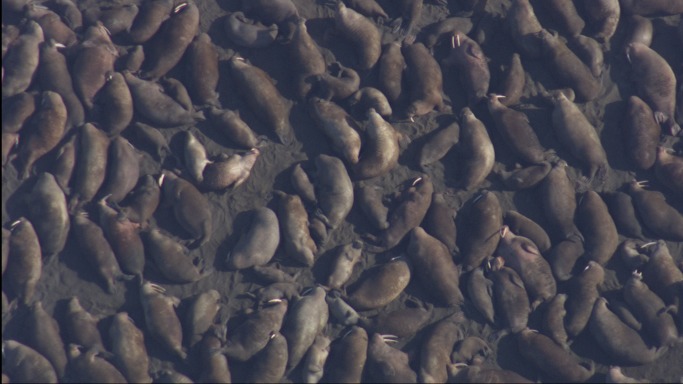
Example Cineflex image taken at an altitude of approximately 915 m. Image of Pacific walruses hauled out on the northwest coast of Alaska on 25 August 2011. Image is projected at 100%, though video and reference image resolution allowed tusk∶snout measurements to be made at 400% or more digital zoom. Pixel size is ∼0.9 cm.

**Figure 3:**
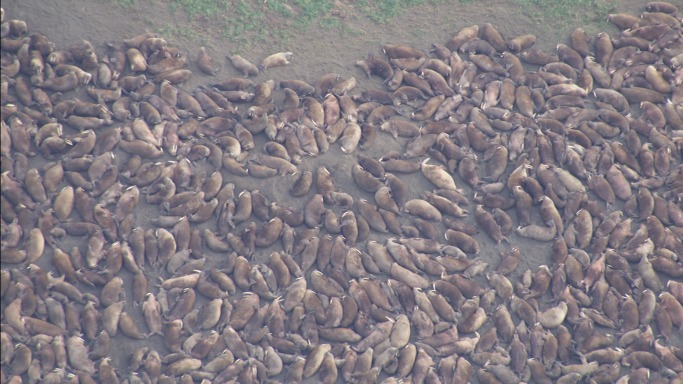
Example Cineflex image taken at an altitude of approximately 1220 m. Image of Pacific walruses hauled out on the northwest coast of Alaska on 26 August 2011. Image is projected at 100% though video and reference image resolution allowed tusk∶snout measurements to be made at 400% or more digital zoom. Pixel size is ∼1.3 cm.

Based on the scaled transect images, we determined the pixel size for images exported from the Cineflex video within the Image-Pro Plus software. The area covered by a pixel ranged from ∼0.9 cm at 915 m (Fig. 2) to ∼1.3 cm at 1220 m (Fig. 3). Our computer screen resolution of video images taken from within this altitude range allowed aging and sexing of individuals. We were able to positively identify 19 males (<1% of total walruses examined) because of the presence of a bacular ridge or skin bosses typical of older adult males [1]. The majority of individuals were females. However, the herd likely included some number of young adult males, which are often indiscernible from adult females.

Out of the 2,140 randomly selected walruses, we were able to classify 2,030 to either an 8-category or 3-category age class (95% of selected). Of the females, we classified 1,329 (66%) to the 8-category age resolution and an additional 682 (34%) to the 3-category age resolution (Table 3). The age structure of that portion of the sample that could be determined to the 8-category level differed from that portion that could only be determined to the 3-category level (*x^2^_2 = _*85.00, *P*<0.01), primarily because of the large number of calves and yearlings that could not be distinguished from each other. This resulted in a much smaller proportion of 0–2 yr-olds in the 8-category classification (Table 3). The proportion of 0–2 yr-old walruses that could be classified to the 8-category level (52%) was substantially less than the proportions of 3–5 or 6+ yr-olds that could be classified to that level (68% and 74% respectively, *t*_2027_ <−4.7, *P*<0.01).

**Table 3:** Numbers of female and young (known males removed) Pacific walruses in each age class based on 8-category and 3-category classifications of samples from herds hauled out on the northwest coast of Alaska in fall of 2010 and 2011.

	Classification resolution					
	8-category		3-category		3-category	
Age (years)	No.	#/100 adults	No.	#/100 adults	Total	#/100 adults
0	204	25	.	.	**.**	**.**
1	26	3	.	.	**.**	**.**
2	94	12	.	.	**.**	**.**
Subtotal 0–2	324	40	300	103	**624**	**57**
3	44	5	.	.	**.**	**.**
4–5	155	19	.	.	**.**	**.**
Subtotal 3–5	199	24	92	32	**291**	**27**
6–9	258	.	.	.	**.**	**.**
10–15	232	.	.	.	**.**	**.**
15+	316	.	.	.	**.**	**.**
Subtotal 6+	806	---	290	---	**1096**	**---**
Total	1329	---	682	---	**2011**	**---**

In 2010, we visually classified 460 walruses with 230 (50%) aged at the 8-category level and 230 at the 3-category level. We did not record “unknown” ages in 2010. In 2011, we visually aged the first 267 walruses, and classified 94 (35%) and 155 (58%) to 8-category and 3-category age-classes respectively with 18 (7%) classified as “unknown”. Once we began measurement-based aging, we classified an additional 1413 individuals and found images suitable for valid tusk length and snout dimension measurements for 650 (46%), which all provided 8-category ages (Table 4). Another 306 individuals (22%) did not present head profiles adequate for valid measurements, but did provide views with enough visual cues to estimate age although 63% were at the lower 3-category resolution (Table 4). Another 365 (26%) were small calves that could be aged accurately without measurements. Ninety-two (6%) were larger individuals (≥3 yrs) that provided no view of their tusks and snout and their age was classified as “unknown” (Table 4).

**Table 4:** Numbers of walruses selected for measurement-based aging and resolution of age classifications from hold sequences recorded in 2011.

Age-class resolution	Measureable images^1^	No. aged visually^1^^,^ 2	No. aged on body size^3^	No. classified as “unknown”^1^^,^ 2
	650 (0.46)^a^	306 (0.22)^a^	365 (0.26)^a^	92 (0.06)^a^
8-category	650 (1.00)^b^	113 (0.37)^b^	196 (0.54)^b^	---
3-category	0 (0.00)^b^	193 (0.63)^b^	169 (0.46)^b^	---

a Proportions in parenthesis are relative to 1,413 attempted measurements.

b Proportions in parenthesis are relative to the column totals given in the 1^st^ row.

1 Column only includes larger animals where aging required tusk and snout measurements.

2 An attempt was made to find a measurable image but none with the requisite perfectly perpendicular front or side view was found, and only a visual estimate of age was made or age was classified as unknown.

3 Column only includes smaller animals where aging did not require tusk or snout measurements.

In 2011, we recorded whether age was determined in the reference image for 1,543 individuals in 39 of 40 hold sequences. We aged 762 (49%) in the reference image while the other 781 (51%) could only be aged by searching the remaining hold sequence for an age-able head profile. We found measurable head profiles more often when searching hold sequences, with 39% and 45% of final ages based on measurements for reference and non-reference images respectively. For animals aged in non-reference images, an average of 15 sec (std. dev. = 12 sec, max = 82 sec) of video separated the reference image from the eventual age-able image with 95% of ages assigned in ≤40 sec either side of the reference frame.

**Figure 4:**
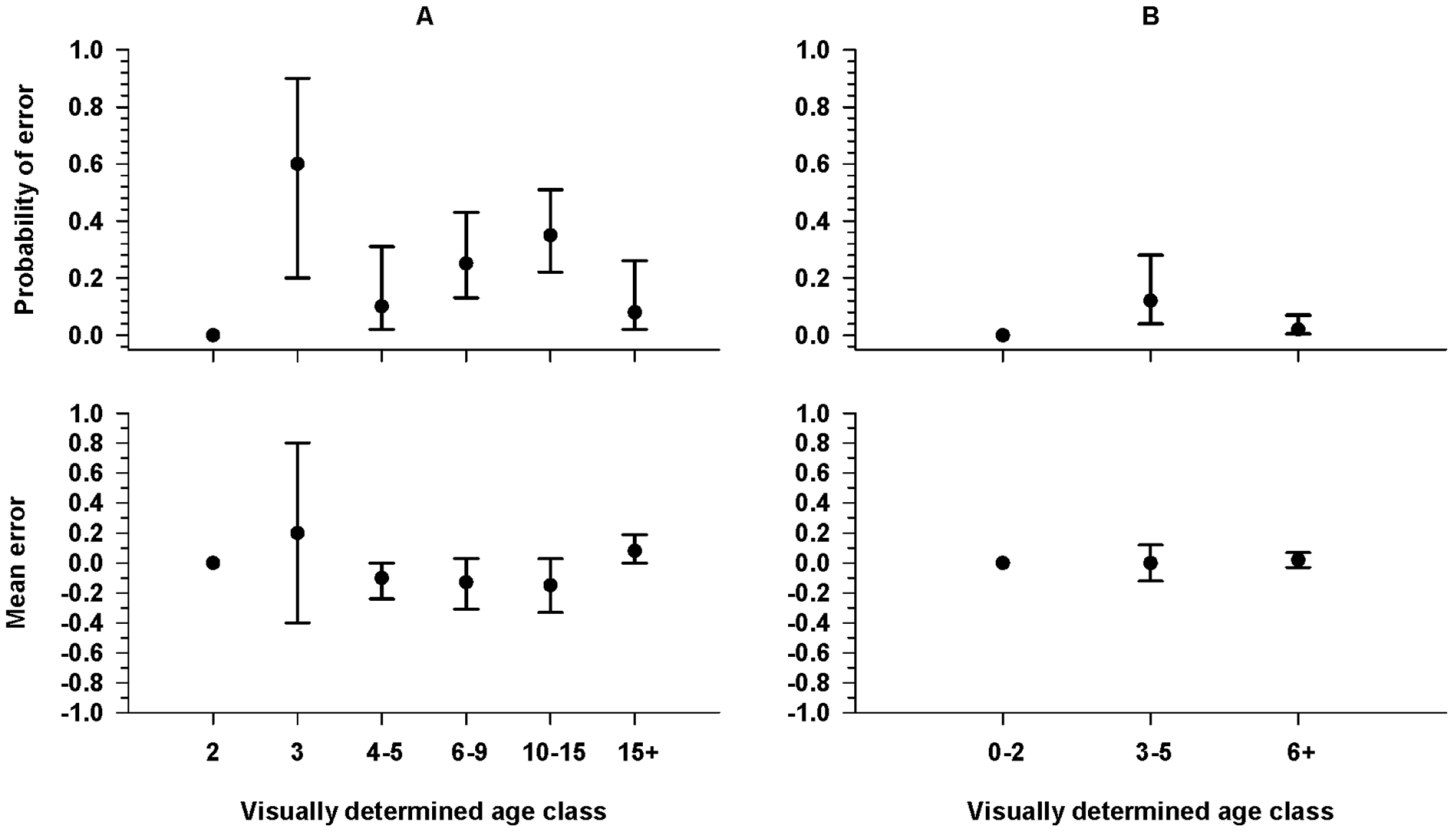
Probability of an error and mean size of the error in visual age classifications. Probability of error and mean size of error for the 8-category (A) and 3-category (B) visual-based age classifications relative to measurement-based classifications of Pacific walruses hauled out on the northwest coast of Alaska in fall of 2010 and 2011. Measurement-based age classifications are not possible for 0- and 1-yr classes, because tusks are not visible from walruses in these age classes. Error bars indicate 95% confidence intervals.

For walruses with both visual and measurement-based age determinations, 22% (29/134) of the 8-category and 4% (6/152) of the 3-category classifications disagreed. There were no differences between visual and measurement-based classification of age class 2 (8-category) or age class 0–2 (3-category) walruses, but the mean probability of error in the visual estimates of other age classes ranged from 0.05 to 0.20 for 8-category classifications and from 0.01 to 0.06 for 3-category classifications (Fig. 4). However, none of the determinations differed by more than one age class, and for most age classes, the visual determination was too high in some cases and too low in others, so that the mean error was always less than one age class (Fig. 4).

**Figure 5:**
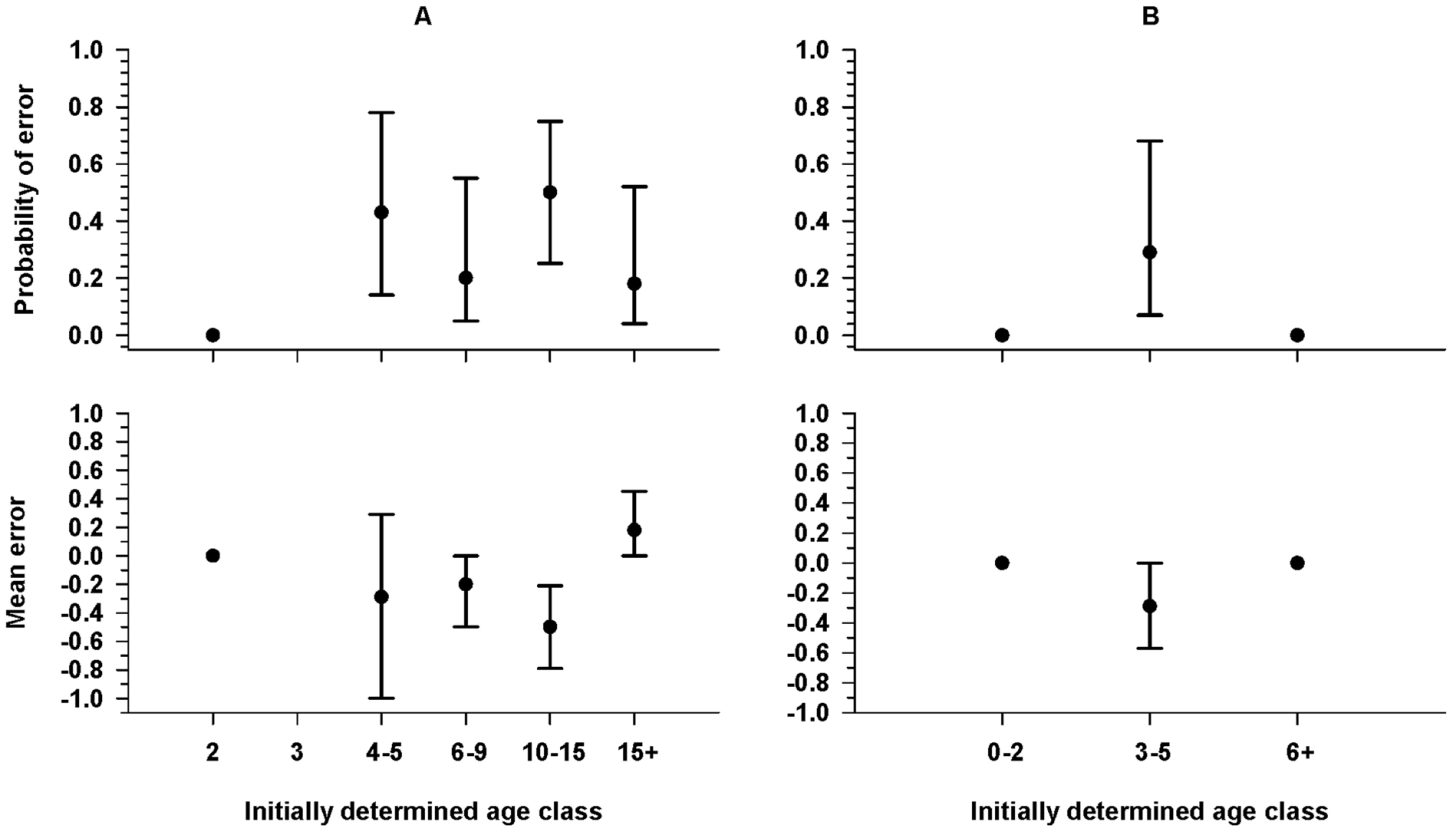
Probability of an error and mean size of the error in measurement-based age classifications. Probability of error and mean size of error for the 8-category (A) and 3-category (B) measurement-based age classifications relative to a second “better” measurement-based classification of Pacific walruses hauled out on the northwest coast of Alaska in fall of 2010 and 2011. Measurement-based age classifications are not possible for 0- and 1-yr classes, because tusks are not visible from walruses in these age classes. Error bars indicate 95% confidence intervals.

For walruses with two measurement-based age determinations, 33% (14/43) of the 8-category and 5% (2/43) of the 3-category classifications disagreed. Again there were no differences between the two determinations for age class 2 (8-category) or age class 0–2 (3-category) walruses. The mean probability of an error in the initial determination of other age classes ranged from 0.18 to 0.50 for 8-category classifications (Fig. 5). The mean probability of an error in age class 3–5 (3-category) was 0.29; there were no differences between the two determinations for age class 6+ (Fig. 5). Age class determinations differed by as much as two age classes, but again the initial determination was too high in some cases and too low in others, and the mean error was always less than one age class (Fig. 5).

### Spatial and temporal distribution

**Figure 6:**
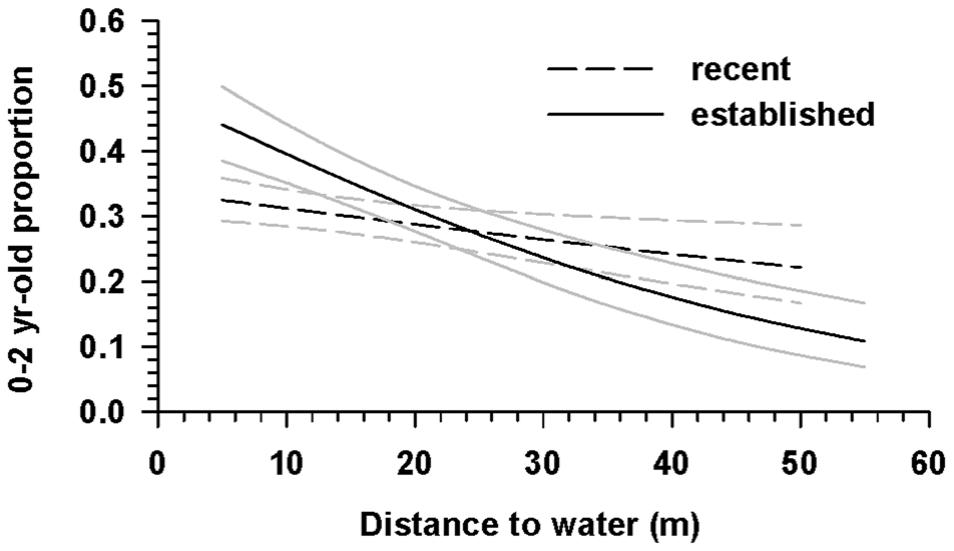
Proportion of 0–2-yr-olds as a function of distance to water within Pacific walrus herds hauled out onshore near Pt. Lay, Alaska. Within herd age-structure relative to distance to water for Pacific walrus herds hauled out on the northwest coast of Alaska in September 2010 and August 2011. Curves are proportion of 0–2-yr-olds for locations that have been occupied for less than a day (dashed) or more than a day (solid). The gray lines denote 95% confidence intervals.

We did not detect differences in proportions of age class 0–2 walruses among herds or dates (*F*_7, 40_ = 1.10, *P* = 0.38). The relative number of age class 0–2 walruses tended to decrease with distance from the water (Fig. 6). The rate of decrease depended on how recently the area had been occupied (interaction *F*_1, 1979_ = 7.35, *P* = 0.01), with mean proportion of age class 0–2 walruses decreasing from about 0.32 to 0.22 in areas that were occupied for less than a day (*F*_1, 1979_ = 32.80, *P*<0.01) and from about 0.44 to 0.11 in areas that were occupied for more than a day (*F*_1, 1979_ = 6.63, *P* = 0.01, Fig. 6).

### Herd size and density

Individual herds occupied from 9,302 to 21,611 m^2^ of beach, with estimated total numbers of walruses ranging from 8,300 to 19,400 (CV 0.03–0.06, Table 5) per herd. Density of walruses was essentially the same within each herd on each day (Table 5, *F*_3,76_ = 0.05, *P* = 0.98). The combined ratio density estimate based on data from all herds and days was 0.88 walruses/m^2^ (std. err. = 0.02). The areal coverage of the combined herds (Fig. 1) indicates 19,500 (±999) walruses were hauled out on 25 August 2011 and 32,900 (±1,639) on 26 August 2011 (Table 5).

**Table 5:** Estimates of overall density and total number of walruses in herds hauled out on the northwest coast of Alaska in fall of 2011.

Herd	Day	Area occupied by herd (m^2^)	Total grid cells^1^	Walrus density^2^ (Std. Err.)	Total no. of walruses (Std. Err.)
North	25 Aug.	9,302	109	0.89 (0.03)	8,300 (289)
South	25 Aug.	12,613	149	0.89 (0.06)	11,200 (710)
North	26 Aug.	15,766	162	0.86 (0.04)	13,500 (654)
South	26 Aug.	21,611	217	0.90 (0.05)	19,400 (985)

1 Total number of 10 m×10 m grid cells required to cover the herd. Estimates are based on a random sample of 20 of these cells from each herd on each day.

2 Walruses/m^2^.

## Discussion

### Filming and marking

Our aerial videography method acquired overhead HD video images from altitudes of 915 m to 1,220 m that were suitable for estimating the age structure of walruses with little disturbance to the herd. Generally, light onshore winds combined with little background surf noise required the higher flight path while an offshore wind direction in combination with higher surf allowed lower flight paths. Images exported from Cineflex video footage taken at altitudes of 915 m and 1,220 m had pixel sizes of ∼0.9 cm to ∼1.3 cm respectively. This was possible because of the 42× to 84× optical zoom capability of the Cineflex video system held steady by its computer controlled gyro-stabilization system. At 915 m, the image resolution allowed detailed observations – e.g., seeing the bacular ridge of males lying on their backs, and tusks of individuals ≥2 yrs old were easily measurable (Fig. 2). However, even with a perfect view, tusks of 1-yr-olds barely protrude below the upper lip (see Figure 1 in [24]), and are rarely measurable. At 1,220 m, image resolution is not as detailed but still adequate to measure tusk lengths of 2-yr-olds (Fig. 3).

Video footage has an advantage over still camera photos primarily because it takes multiple images per second for an extended time, which increases the probability of obtaining a measurable image for estimating the age of any randomly selected walrus. In addition, viewing video frame-by-frame makes judging when a walrus has presented a measureable head profile much easier than when only single images are available. However, 1080p HD video images are of lower resolution than images taken with a good quality still camera at the same optical magnification. For example, HD video (1920×1080) has 2 Mp per frame, and a frame spanning 50′ would have 1920/300″ = 3.2 pixels/inch or a pixel size of 0.8 cm. A still camera taking high quality images (4743×3162) has 15 Mp per frame, and a frame spanning 50′ would have 4743/600″ = 7.9 pixels/inch or a pixel size of 0.3 cm. In our situation, only a gyro-stabilized camera system could provide <1 cm pixel sizes from nearly 1 km distance aboard a vibrating helicopter (Video S1). However, if only standard camera systems are available, a still camera has the advantage of allowing greater digital magnification of images, though many sequential images (e.g., 10+ seconds at 2+ frames per second) should be collected to increase the probability of obtaining images with measureable head profiles. It may be possible to acquire adequate imagery from a lower altitude/elevation with less expensive, traditional HD video technology or with a still camera system and without disturbing the herd at haulout sites with favorable topography (e.g., cliffs or higher beach over-looking a haulout site). The topography along the northwestern coast of Alaska is flat, precluding this approach in our study area. However, on the Chukotka coast of Russia where there are cliffs above some of the beaches where walruses are hauling out, it may be possible to use traditional HD videography or still cameras to acquire the images needed to estimate the age-structure of the herds.

A computer controlled, gyro-stabilized camera system has the advantage of allowing acquisition of steady images at ultra-high optical zoom from moving platforms. These systems can be mounted on any type of vehicle and could potentially be used in many situations where detailed observation from a distance is advantageous. For example, they may be useful for making long distance ground-based observations of cryptic, skittish species such as large cats, wolves or other top predators. They could also be mounted on fixed-wing aircraft and used to collect traditional age-structure information from herd-forming ungulates that can stampede when disturbed (e.g., caribou or various African ungulates). To date, computer controlled, gyro-stabilized camera systems have been employed primarily to make “Nature” type documentaries. We demonstrate here that they can also be a valuable research tool.

We found use of ground reference markers to scale transect images was simple and enabled us to get reasonable measures of herd density. However, we recommend that future surveys should modify the camera system to record the information needed to scale the images based on optical characteristics, so that ground markers would not be required. Photogrammetrically scaled images would allow all images (including hold images) to be used to make morphometric measurements (e.g., [38]), and allow easier separation of the 0–2 age classes in particular.

### Age classification

We were able to classify ∼95% of the randomly selected walruses to at least the 3-category level, suggesting that, at this level, there would be little bias in estimated age structures due to the inability to find adequate images. In this case, we found 57 0–2 yr-olds and 27 3–5 yr-olds per 100 adults after removing the known adult males (Table 3). However, the proportion that could be classified to the 8-category level varied with age, with only 52% of the 0–2 yr-olds classifiable at this level compared to 68% and 74% of the 3–5 and ≥6 yr -olds (Table 3). This suggests that there may be bias in age structures estimated to the 8-category level. In particular, 1-yr-olds appear most underrepresented in the 8-category age distribution with only 3 per 100 adults (Table 3) because 1-yr-olds were more likely than other age classes to be classified at the 3-category level. This is because 0- and 1-yr-olds are classified on relative body size rather than tusk and snout measurements (i.e., no or barely visible tusks; see Figure 1 in [24]). The very small size of calves appears to make them readily identifiable as we found 25 per 100 adults, which is high compared to at-sea surveys [24]. Similarly, 2-yr-olds have a small tusk that makes them easily identifiable and we found 12 per 100 adults. However, the constantly changing scale of the hold sequence video images made definitively judging the relative size of 1-yr-olds difficult. We could reduce or eliminate the bias that results from an inability to distinguish 1 yr-olds in future surveys by using photogrammetrically scaled images allowing measurements of actual body lengths, and make 0- and 1-yr-old classifications an objective measurement-based process similar to aging animals with tusks.

Previous at-sea age-structure surveys employed visual classifications (as opposed to measurement-based) and suggest accuracy can vary among observers with misclassification errors particularly high for inexperienced observers [23], [24]. However, previous at-sea surveys did not utilize photo documentation, age classifications could not be verified, and true classification error rates could not be determined. We found visual age misclassifications were minimal at the 3-category level with the probability of error just over 0.1 (and unbiased) for 3–5 yr-olds and ≤0.02 for the other two age classes. However, the probability of error was >0.2 for three of six measurable age classes at the 8-category level although mean size of the error was small and mostly unbiased. This type of error can be eliminated by using only measurement-based classifications from images, which would also result in greater precision in estimates of the proportional abundance of walruses in each age class.

Video or still image documentation has several advantages over real-time visual classifications. For example, imagery can be examined frame by frame and replayed allowing time to make accurate classifications. In addition, measurement-based classifications are repeatable and verifiable even when using inexperienced observers. We did not examine the effect of observer in this study, but the precision attainable with digital measurements of high-resolution images assures that multiple observers will rarely classify the same animal differently when examining the same image. In addition, if threshold values used to separate age-classes change due to additional age specific data on tusk∶snout measurements or a different definition for how the threshold value is determined, new age ratios based on the new threshold values could easily be determined. In contrast, real-time age determinations can never be reassessed.

Measurement-based classifications have different sources of error. Specifically, they depend on obtaining images of walruses that are perpendicular to the long axis of the walrus's tusk to enable accurate measurements of tusk length. In this study, we mimicked having only one image available (the reference image) and after determining that an image was measureable, we examined the rest of the video for a “better” image and found that, for 8-category classifications, the probability of error was ≥0.2 for the 4–5, 6–9, and 10–15 yr-old age classes with a bias toward classifying too low (Fig. 5). This bias occurred because even a slightly oblique camera angle can create an apparent shortening of the tusk. Even for the 3-category classification, the probability of a negative error was ∼0.3 for walruses initially classed as 3–5 years old. This type of error has the potential of slightly over-estimating 3–5 yr-old age ratios; however, underestimating the age of 10+ yr-old adults will have no effect on juvenile age-ratios expressed as number per 100 adults (i.e., 6+ yrs). This type of error is due to observer ability to judge the adequacy of the facial profile presented in an image, and will likely vary by observer, level of experience and the number of images from which to choose. Under the single-available-image scenario tested here, we found a better image ∼50% of the time. In contrast, images chosen from the video sequence are less prone to this bias because frame-by-frame comparisons make image choice much greater and judgments of correct head profile much easier. Overall, to obtain more objective, precise and verifiable estimates of the proportion of walruses in each age class, it would be better to use photo documentation and measurement-based classifications rather than real-time visual classifications.

An additional source of error in estimating age of walruses from tusk∶snout ratios is individual variation in tusk growth rates. In particular, tusk ratios of 6–9 yr-olds overlap those of 4–5 yr-olds by approximately 47% for snout width and 50% for snout depth (see Table 1 in [23]). This type of aging error is unavoidable, and occurs whether age classifications are based on visual estimates or measurement-based estimates. This type of misclassification increases variability in estimates of age class proportions, but may not bias the estimates if misclassifications in either direction are equally likely. In addition, the bias introduced by classifying even up to 50% of the 4–5-yr-olds as adults will be small because there are so few 4–5-yr-olds in comparison to the number of adults in the population [24].

### Spatial and temporal distribution

We found that dependent walruses (0–2 yr-olds), presumably with their mothers, tended to be distributed at locations within the herd closer to water and that this tendency became more pronounced as the herd spent more time on the beach (Fig. 6). The practical implication is that unbiased estimation of herd age-ratios will require either a random sample of images from the entire herd, or an approach that explicitly accounts for the spatial and temporal structuring. Consistent with this recommendation, Fay et al. [22] noted a tendency for immature animals and females with calves to be most numerous along the periphery of very large herds lying on shore, and thus excluded age ratio data from groups with incomplete classification to avoid biasing age ratios in favor of younger age classes. The age-related structuring of the herds also suggests that randomly sampling walruses from the edge of the herd for other purposes (e.g., tagging, biopsying) will not be representative of the age structure of walruses in the herd.

The propensity for females with dependent young to distribute more closely to the water may simply reflect the difficulty calves have moving deeper into the herd. Alternatively, females with dependent young may be deliberately minimizing their “escape” distance to the water, and thereby reduce their calves' trampling risk. As a strategy to mitigate calf-trampling risk, herd structure may have resulted from recent experiences the current population has had with mass disturbance events [1], [25], [26], [31], [32], or it may reflect an adaptive behavioral response that has developed over evolutionary time-scales.

### Herd size and density

Although our study focused on coastal haulouts in only a very limited geographical area during a 2-year period, the densities we observed were remarkably consistent at about 0.88 walruses/m^2^ among herds and dates. This density is the same as the 0.882 walruses/m^2^ used by Kochnev [27] to estimate numbers of females and calves hauled out on Wrangel Island beaches in the 1990's and at Cape Serdtse-Kamen in fall 2009 [39]. Fay and Kelly [25] cited a slightly lower density estimate of 0.83 walruses/m^2^ as the maximum for herds of mainly females and young when estimating herds of up to ∼52,000 walruses hauled out on the beaches of St. Lawrence Island and the Punuk Islands in the autumn of 1978. Thus, observed herd densities have been similar despite differences in locality and herd size. Within the herd, our range of densities (0.40 to 1.49 walruses/m^2^) was somewhat greater than the most probable range of variation (0.56 to 0.62 walruses/m^2^) used to estimate numbers of walruses hauled out at St. Lawrence Island [25].

In general, walruses are believed to haul out in dense shoulder-to-shoulder groups as a defense against predators [1]. Forming aggregations is a common anti-predation strategy [40], but for walruses, group size likely reflects a balance between predation risks and trampling risk. On sea ice, this balancing of risks may explain why cows with calves are more common in medium size than smaller or larger groups [23] with calf∶cow ratios peaking for groups of ∼40 animals [24]. On land, walruses appear to favor forming a few very large groups rather than the alternative of forming many small groups spread out along the coast. Presumably, this is also an anti-predation strategy; however, this strategy comes at the cost of increased trampling risk.

### Use of age data

Age-structure information can be used in combination with other data to make inferences about demographic parameters. For example, age-structure data can be combined with information on population growth rate or fecundity to estimate survival rates [41], and can be incorporated into population models to project population dynamics [14], [42]. Parameters such as reproductive and juvenile survival rates likely undergo density dependent changes, making them potential indicators of population status relative to carrying capacity, and can be informative to management decisions [43].

Age ratios by themselves are often used to make inferences about recruitment rates in populations where the number of adults can be assumed to remain relatively constant. In this context, age ratios (e.g., calf∶cow ratios) have been used extensively as an index of population productivity for ungulate populations [15]–[18]. Similarly, age ratios have been used to infer annual recruitment in whales [44], [45] and pinnipeds including fur seals [19] and southern elephant seals [20], [46]. The Fay [22], [23] classification scheme has four single year juvenile age classes (0, 1, 2, and 3 yr-olds) and one mixed year juvenile age-class (4–5 yr-olds). In effect, this extends the temporal record of productivity and juvenile survival available in the age ratio data in contrast to ungulates, which are nearing adult size at one year of age so that only calves of the year are identifiable in age ratio surveys. Thus, the 8-category age structure is the most demographically informative, and it will be important to improve our ability to distinguish 1-yr-olds in future surveys to maximize the potential usefulness of the age structure information. In addition, we will need to implement a sampling design that accounts for the within herd spatial and temporal age structure documented here. Finally, if we are to make population level inferences from coastal haulout age ratios, we will need to determine if haulout probabilities differ by age or reproductive status, which could be accomplished with current satellite tagging technology [11], [47]. The calf ratios documented here are ∼50% higher than the highest calf ratio documented during at-sea surveys in the 1980's and 1990's [23], [24], but we do not yet know if this reflects an increase in productivity, differences in haulout probabilities, or sampling error (e.g., over-sampling near the waterline).

Walruses are expected to continue to increase their use of coastal haulouts with decreasing availability of summer sea ice. Aerial videography at these haulouts may provide an effective approach for estimating the population age structure. This type of age information will likely prove essential for understanding walrus population dynamics in a changing Arctic environment.

## Supporting Information

Video S1 **Example Cineflex video sequence taken at an altitude of approximately 915 m.** Video sequence of Pacific walruses hauled out on the northwest coast of Alaska on 25 August 2011. Reference and other aging images where exported from similar video sequences. Video resolution allowed tusk∶snout measurements to be made at 400% or more digital zoom. Pixel size is ∼0.9 cm.
(MPG)
